# Reciprocal relations between past behavior, implicit beliefs, and habits: A cross-lagged panel design

**DOI:** 10.1177/13591053231164492

**Published:** 2023-04-19

**Authors:** Kyra Hamilton, Daniel J Phipps, Natalie J Loxton, Kathryn L Modecki, Martin S Hagger

**Affiliations:** 1 School of Applied Psychology, Griffith University, Brisbane, Australia; 2 Menzies Health Institute Queensland, Griffith University, Gold Coast, Australia; 3 Health Sciences Research Institute, University of California, Merced, Merced, United States of America; 4 Psychological Sciences University of California, Merced, Merced, United States of America; 5 Faculty of Sport and Health Sciences, University of Jyväskylä, Jyväskylä, Finland

**Keywords:** binge drinking, habit, implicit association test, implicit beliefs, university student

## Abstract

The current study assessed cross-lagged relationships between binge drinking, implicit beliefs, and habit in undergraduate university students (*N* = 105). Students completed self-report survey and implicit measures in lab visits 3 months apart. A structural equation model revealed cross-lagged relations between habit and behavior, and some evidence for a reciprocal relationship between implicit beliefs and habit. Implicit beliefs were related to alcohol behavior across time, but no cross-lagged relationship was observed. Findings provide preliminary support for recent advances in habit theory, suggesting that implicit beliefs and habit may develop in tandem or even share common knowledge structures and schemas.

## Introduction

Binge drinking in young adulthood has been associated with numerous negative outcomes, including both short term alcohol related injury or risk taking (Isralowitz and Reznik, 2015; Karagülle et al., 2010), and increased risk of long term damaging health consequences and substance abuse (Åberg et al., 2017; Chassin et al., 2002; Viner and Taylor, 2007). As a result, numerous researchers have attempted to formulate strategies and programs with the ultimate goal of reducing this dangerous behavior. However, the development of strategies to inform behavior change intervention necessitates a fundamental understanding of the determinants of the target behavior and associated processes (Hagger et al., 2020). Specifically, binge drinking has been theorized as an “appetitive” behavior influenced by factors that reflect implicit, non-conscious processes that determine behavior automatically and without the need for excessive conscious input (Caudwell et al., 2019; Wiers et al., 2007). Importantly, the automatic nature of these processes means they are theoretically likely to impact behaviors like binge drinking regardless of one’s own intention or knowledge of the health risks associated with the behavior (Stacy and Wiers, 2010). Thus, the examination of constructs that represent these non-conscious determinants of behavior is considered important to develop more comprehensive models of behavior which may account for a wider range of behavioral determinants. These constructs, representing automatic pathways to behavior, have been shown to successfully augment traditional social cognition theories such as the theory of planned behavior (Ajzen, 1991) and health action process approach (Schwarzer and Luszczynska, 2008), leading to integrated dual process models of behavior (Caudwell et al., 2019; Hagger et al., 2017; Phipps et al., 2021, 2022b).

Two key psychological constructs that reflect non-conscious processes in such theories are implicit beliefs and habits. Implicit beliefs as a construct cannot be directly measured and are instead inferred from indirect measures of association such as reaction time tasks or priming effects, rather than traditional self-reported scales (De Houwer et al., 2009). These measures proport to reflect individuals’ automatically activated cognitions and schemas which are theorized to be developed through repeated experiences and evaluations of a behavior, such as alcohol consumption. Once developed, these implicitly associated evaluations or information are then triggered automatically upon encountering behavior related stimuli, thus effecting future behavioral occurrences. Another construct which makes up the automatic drivers of behavior is habit, defined as behavioral sequences which are enacted with little or no forethought upon encountering a behavior related stimulus (Gardner et al., 2012; Hagger, 2020; Mazar and Wood, 2018), likely developed through frequent experience with the behavior in the presence of stable contexts (e.g. in a bar) and cues (e.g. seeing others drinking) (Orbell and Verplanken, 2010). For the most part the habit construct is tapped via meta-cognitive self-reported measures where individuals reflect upon the extent to which they perceive their behavior as enacted without conscious thought, efficiently, and automatically. Both implicit beliefs and habits have been shown to directly predict behavior and add unique variance over and above other social cognition constructs from social cognition theories and past behavior (Brown et al., 2020; Phipps et al., 2020), including research on binge drinking behaviors in university students (Hamilton et al., 2020).

However, theory-based tests of the relationships between constructs representing non-conscious processes like implicit beliefs and habits have relied heavily on cross-sectional or prospective designs. That is, designs in which all data is collected at a single timepoint, or only a key outcome is measured at a later date. Such approaches assume a recursive-chain causal structure underlies relationships among theory constructs (Shingles, 2009). This assumption may oversimplify the relationship between constructs and neglects the potential for stability and reciprocal relationships among constructs, which necessitates the adoption of panel designs where all constructs are measured at multiple time points. Research on social cognition theories, like the theory of planned behavior (Ajzen, 1991), has applied panel designs to study stability and reciprocal relationships among theory constructs (Hagger et al., 2001; Liska et al., 1984). Such designs, however, have seldom been applied to examining relations among constructs that reflect implicit, automatic processes, such as implicit beliefs and habits. Although many studies have regressed habit and implicit beliefs on subsequent behavior, few have tested the association between past behavior and these constructs (Forscher et al., 2017; Lindgren et al., 2016, 2018, 2023; Paige et al., 2022), and none to date have explored reciprocal relationships between implicit beliefs and habits.

This is in spite of theory suggesting that such relations may exist. Specifically, theory suggests that previous behavioral experience may maintain implicit beliefs and habits over time. For example, habit theory suggests that habits are developed through repeated experience in stable contexts (Gardner and Lally, 2018). Past behavioral frequency may, therefore, serve as a proxy for such experiences and be expected to predict habit. In addition, it might also be reasonable to speculate that habits maintain implicit beliefs over time. This is because individuals with stronger habits are more likely to have consistently experienced the behavior coinciding with positive evaluations of the behavior (Hagger, 2020). In contrast, enacting behavior in the absence of habit is less likely to be perceived as in line with one’s goals and sense of self (Gardner and Lally, 2013), and more likely to be perceived as effortful (Ouellette and Wood, 1998; Verplanken and Melkevik, 2008). Over time, this is likely to have developed into strong implicit beliefs with respect to the behavior. Similarly, positive implicit beliefs may aid in the activation and utilization of the cue-behavior scripts which underlie habitual responding (Hagger, 2020), while negative implicit beliefs have been theorized to inhibit behavior even in the presence of cues (Brand and Ekkekakis, 2018), potentially reducing the likelihood of habit formation and habitual actions. This points to the possibility that habits and implicit beliefs may develop in tandem and each serve to mutually sustain or reinforce each other. However, to date, little research has provided evidence for these propositions and there is need for studies examining reciprocal relations between implicit beliefs, habits, and behavior.

We aimed to fill this evidence gap in a two-wave cross-lagged panel design on a sample of Australian undergraduate students enrolled in a first year course, given the high rates of binge drinking in this sample and the notable change in drinking patterns often observed in first year students (Cleveland et al., 2012; Hallett et al., 2012). Such designs permit formal assessment of reciprocal relationships while controlling for covariance stability in constructs over time, which cannot be tested in cross-sectional or prospective designs (Marsh et al., 2006). We hypothesized positive and non-trivial relationships between habits and implicit beliefs measured on an initial occasion (T1) and behavior measured on a second occasion (H1 and H2, respectively), 3 months later (T2). We also hypothesized positive, non-trivial relationships between behavior at T1 and habits (H3) and implicit beliefs (H4) at T2, consistent with previous research (Hamilton et al., 2020). In addition, we expected positive and non-trivial cross-lagged relationships of habits at T1 with implicit beliefs at T2 (H5) and implicit beliefs at T1 with habits at T2 (H6), consistent with theory on implicit effects on behavior (Hagger, 2020).

## Methods

### Participants and procedure

Participants were undergraduate university students of legal drinking age recruited using the psychology first-year subject pool, flyers, and social media posts as part of a larger ongoing lab-based longitudinal study. Undergraduate students aged 18–25 years, reporting consuming alcohol socially, and not currently pregnant were eligible for inclusion. Participants were offered course credit or a coffee voucher and entry into a prize as an incentive for participation. A total of 299 undergraduate students completed the baseline measurement battery. However, 189 did not attend the follow-up lab session 3 months later, and 5 participants met the exclusion criteria for the IAT scoring at either time point (Greenwald et al., 2003). Thus, the final sample consisted of 105 students (M_Age_ = 19.82, SD_Age_ = 2.36; 71 Female, 34 Male). Sample information is presented in Table 1. Despite the notable attrition, analysis indicated participants who returned for T2 measurement did not differ from those who completed the follow up in terms of age (t(297) = 0.80, p = 0.425), gender (χ^2^(1) = 0.37, *p* = 0.541), ethnicity (χ^2^(5) = 2.42, *p* = 0.789), employment status (χ^2^(4) = 4.82, *p* = 0.306), or relationship status (χ^2^(1) = 0.58, *p* = 0.448). Further, there were no significant differences between those who completed T2 and those who did not on study variables at T1 (Wilks’ λ = 0.998, F(3, 288) = 1.15, p = 0.331).

**Table 1.** Demographic information for the final sample and sample at baseline.

Statistic	Baseline sample	Final sample
Mean age (SD)	19.71 (2.36)	19.82 (2.36)
Gender		
Female	204	71
Male	95	34
Other	0	0
Relationship status		
Unemployed	8	2
Full-time work	5	1
Part time/casual work	122	36
Full-time student	157	64
Part-time student	7	2
Ethnicity		
Caucasian/White	226	77
Indigenous Australian/Torres Strait Islander	8	4
Asian	31	12
Pacific Islander	2	0
African	7	3
Other	25	9
Marital status		
Not married	298	105
Married	1	0

At T1, participants were asked to read a brief information passage and complete a consent form. After informed consent was given, participants completed the computerized drinking identity IAT administered by the Inquisit™ experimental software followed by an online survey measuring demographic factors and self-report measures of binge drinking habit and binge drinking behavior. To ensure understanding of the target behavior, participants were presented with a passage defining binge drinking based upon the WHO heavy drinking definition (WHO, 2018) (“Binge drinking is consuming more than six standard drinks on a single occasion”) and a pictorial guide providing examples of a standard drink for common alcoholic beverages. Three months later in the next university teaching period, participants came back to the lab and completed identical measures. The 3 month time lag was conceived as implicit beliefs and habit change is often theorized to occur slowly, especially in natural settings (Gardner and Lally, 2018; Rydell and McConnell, 2006). The study was approved by the University Human Research Ethics Committee.

### Measures

#### Implicit alcohol identity

The drinker identity IAT (DI-IAT; Lindgren et al., 2013) was administered on a standard screen and wired keyboard using Inquisit experimental software. Participants completed seven blocks of trials: three familiarization blocks; two test blocks in which alcohol words (e.g. drinker, drunk) were paired with self-referencing words (e.g. mine, myself) and abstinence words (e.g. sober, abstain) were paired with other-referencing words (e.g. them, they); and, two test blocks where pairings were reversed such that alcohol words shared a response key with other-referencing words and abstinence words shared a response key with self-referencing words. Scores were calculated from participant reaction times in test blocks using the *D*-score scoring algorithm for the IAT (Greenwald et al., 2003), where a participant who was faster at responding when self-referencing words were paired with alcohol words would achieve a positive *D*-score, indicating an implicit identification with alcohol. No error penalty was applied as participants were required to correct errors before proceeding (Lane et al., 2007). Trials of over 10,000 ms were not included in *D*-score calculations, and *D-*scores for participants who responded faster than 300 ms to 10% or more of the trials are discarded (Greenwald et al., 2003).

#### Binge drinking habit

Habit was assessed using the 4-item Self-Report Behavioral Automaticity Index (Gardner et al., 2012), adapted from the 12-item Self-Report Habit Index (Verplanken and Orbell, 2003 ): “Having six or more standard drinks on a single occasion is something I. . . do without thinking, do automatically, do without having to consciously remember, start doing before I realise I am doing it”; scored 1 = *strongly disagree* to 7 = *strongly agree*.

*Binge drinking behavior* was assessed with a two-item scale: “Think about the past 3 months. In general, how often did you have six or more standard drinks on one occasion?”; scored 1 = never to 7 = very *often*; and, “Think about the last 3 months. In general, to what extent did you have six or more standard drinks on one occasion”; scored 1 = *I did not* to 7 = *a very large extent*.

### Data analysis

Hypothesized relations among constructs in the proposed cross-lagged model were estimated in a partial least squares structural equation model analyzed using the WarpPLS v6.0 analysis package (Kock, 2018). Scores from the IAT and responses to survey measures were used as indicators of latent variables representing each model construct in the structural equation model. All paths were set as linear and standard errors were calculated using the “Stable 1” method. A statistical power analysis indicated a minimum sample size of 77, assuming medium effect sizes and power set at 0.80. Data files and analysis scripts are available online: https://osf.io/fejxu/.

## Results

**Figure 1.**
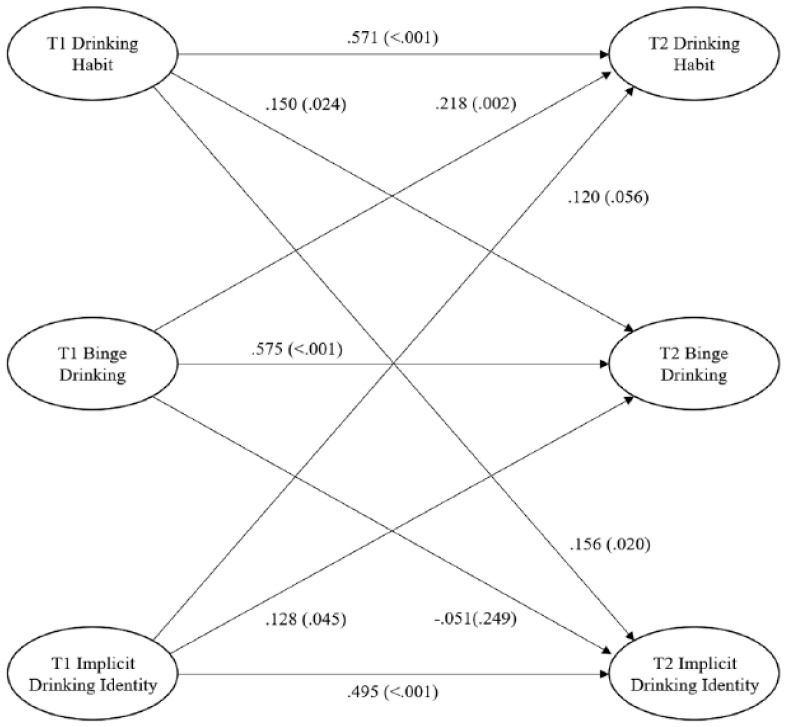
The cross-lagged model of habit, implicit drinking identity, and binge drinking. *p*-Values presented in parentheses alongside standardized model coefficients.

Descriptive statistics, zero-order correlations, and reliability coefficients are available in Table 2. The model showed good fit to data (GoF = 0.646) and explained substantive variance in binge drinking (*R*^2^ = 0.532), drinking habit (*R*^2^ = 0.600), and implicit drinking identity (*R*^2^ = 0.281) at T2 (see Figure 1). Factor loadings were acceptable (normalized loading >0.50, *p*s <0.001). There were statistically significant, modest-strong relationships of implicit drinking identity (β = 0.495, p < 0.001, *f*^2^ = 0.255), habit (β = 0.571, p < 0.001, *f*^2^ = 0.426), and binge drinking behavior (β = 0.575, p < 0.001, *f*^2^ = 0.408) on themselves over time. Implicit drinking identity (β = 0.128, p = 0.045, *f*^2^ = 0.039) and habit (β = 0.150, p = 0.024, *f*^2^ = 0.085) at T1 had small but significant associations with binge drinking behavior at T2. Binge drinking behavior at T1 was associated with habit at T2 with a modest sized effect (β = 0.218, p = 0.002, *f*^2^ = 0.139), but implicit drinking identity was not (β = 0.120, p = 0.056, *f*^2^ = 0.036), reporting a small sized effect which fell short of the conventional cut-off for statistical significance by a trivial margin. Habit (β = 0.156, p = 0.020, *f*^2^ = 0.035) at T1 had a small but statistically significant relationship with implicit drinking identity at T2, but not with T2 behavior where only a trivial effect size was observed (β = 0.051, p = 0.248, *f*^2^ = 0.009).

**Table 2.** Descriptive, zero-order correlations, and reliability statistics for all constructs at T1 and T2.

Variables		1	2	3	4	5	6	7	8
1.	Age	—							
2.	Gender	−0.201*	—						
3.	T1 Binge Drinking	−.058	−.147	—					
4.	T1 Automaticity	0.045	0.034	0.607***	—				
5.	T1 Implicit Drinking Identity	−0.259**	0.110	0.237*	0.199*	—			
6.	T2 Binge Drinking	−0.191	0.001	0.661***	0.509***	0.281**	—		
7.	T2 Automaticity	−0.002	0.025	0.576***	0.700***	0.277**	0.654***	—	
8.	T2 Implicit Drinking Identity	−0.108	0.029	0.125	0.223*	0.516***	0.113	0.197*	—
Mean		19.82	—	2.90	2.42	0.14	2.60	2.49	0.13
Standard deviation		2.36	—	1.73	1.61	0.53	1.67	1.83	0.47
Reliability		—	—	0.92	0.92	0.80	0.93	0.95	0.80

*N* = 105.

* *p* < .05. **p < 0.01. ***p < 0.001.

## Discussion

Several notable findings emerged from this study which build on, and extend, previous research. First, positive and non-trivial relationships between habits and implicit beliefs (H1) and behavior (H2) were observed, consistent with previous research suggesting that appetitive behaviors, such as binge drinking, are influenced by factors that reflect implicit, non-conscious processes and determine future action beyond individuals’ awareness (Caudwell et al., 2019; Hamilton et al., 2020). Results also supported a non-trivial association between past behavior and habits (H3), consistent with the premise frequent experience with the behavior, often in stable contexts, is one of the key determinants of habit development, and that once a habitual response is developed, the behavioral response is rapidly activated on presentation of the paired context independent of goals or intentions (Orbell and Verplanken, 2010; Wood and Neal, 2007). However, no support was found for an association between past behavior and implicit beliefs (H4). The lack of finding in the current study may be because spontaneous changes in implicit beliefs over time occur relatively slowly, if at all (Sloman, 1996). Thus, the lack of significant relationship could be explained as the study period was insufficient to observe substantive change. Alternatively, implicit beliefs may also be influenced by unmeasured factors other than previous experience, such as perceived social pressures, vicarious learning, or propositional information gained from non-experimental sources such as information provision or evaluative inferences (De Houwer, 2014; Karpinski and Hilton, 2001; Olson and Fazio, 2004; Payne et al., 2016; Wiers et al., 2007). These explanations are largely speculative and future research is needed to elucidate the relationship between past behavior and implicit beliefs.

Current findings provide preliminary evidence of a cross-lagged relationship between habits and implicit beliefs, consistent with theories of habits and implicit beliefs developing in conjunction (Hagger, 2020). We found a positive, non-zero association between baseline habits and implicit beliefs (H5), in line with the hypothesis that habits likely allow for the formation of implicit beliefs by promoting frequent behavior alongside positive evaluations. Specifically, engaging in a behavior habitually likely leads to feelings of mastery and alignment with one’s sense of self (Gardner et al., 2020), and should these feelings occur repetitively, these positive evaluations may become connected to the behavior in associative memory (Hagger, 2020). We also expected implicit beliefs to promote habit formation by encouraging repeated behavior in the presence of cues (H6). However, while this relationship was in the expected positive direction, it fell short of the conventional threshold for statistical significance. We were powered to detect medium sized effects, so this may have been a function of inadequate power to find small effects. Nevertheless, findings at least signal potential reciprocal relations between implicit beliefs and habit, but also demonstrate the need for replication in larger samples. This finding has notable theoretical and practical implications. For example, should implicit beliefs and habit develop in tandem, they may represent interconnected knowledge structures or behavioral schema. This may potentially provide an alternative explanation for the implicit belief by habit interaction effects on behavior observed elsewhere (Conner et al., 2007; Phipps et al., 2022a). Further, findings support suggestions that activated affective responses may be an important pathway by which habit influences behavior (Gardner et al., 2020).

### Strengths, limitations, and future directions

The current study has notable value in the presentation of a novel test of cross-lagged relationships between behavior, implicit beliefs, and habit. The findings in support of a cross-lagged relationship between habit and behavior and between habit and implicit beliefs provide preliminary empirical evidence for recent advances in habit theory (Gardner et al., 2020; Hagger, 2020). These findings may be of particular use in understanding the interplay between habit and implicit beliefs both in terms of the development of these constructs and the way by which these constructs influence behavior. While the current study presented potentially useful findings, it was not without limitations. First, we used a brief self-report measure of binge drinking. While similar measures have shown concurrent validity against non-self report measures (Dollinger and Malmquist, 2009), inaccurate or biased responding may pose a threat to the validity of current findings. Future research should replicate the current study using non-self-report measures of alcohol consumption. Similarly, scores on the IAT are likely imperfect, as the construct is inferred from indirect measures rather than tapped directly (Corneille and Hütter, 2020; Schimmack, 2021), allowing for error or unrelated biases in responding. As such, scores from these measures should be treated with due caution, and replications and tests with alternative implicit measures are likely needed to confirm findings. Second, participants in the current study were undergraduate university students. As dangerous drinking in undergraduates is commonplace (Heather et al., 2011), the current findings have bona fide implications for this high-risk population. Nonetheless, results may not generalize to the wider population or other at-risk groups and should be interpreted accordingly. Thus, replication and extension of the current findings are needed to fully elucidate effects in this potentially important area. Lastly, the current study presented a two-wave design with a modest sample size, which, although useful as a test of concept for the discussed effects, requires more intensive future research to corroborate findings. For example, the two wave design precluded the use of a random intercept model (Mulder and Hamaker, 2021), which have been shown as useful in disentangling within and between person effects in cross-lagged modeling. Similarly, given the notable attrition experienced in the current study, most likely due to the collection of data across multiple university trimesters, the current study was only sufficiently powered to detect modest effect sizes. This may explain the findings of borderline significant effects in the current study, as implicit measures often present smaller effect sizes (Greenwald et al., 2009). Future research may seek to confirm these findings with larger samples and additional measurement points.

## Conclusion

The current study sought to test the cross-lagged relationships between implicit beliefs, habit, and binge drinking in a sample of undergraduate students. A cross-lagged relationship was found between binge drinking and habit, but not between binge drinking and implicit beliefs, while the cross-lagged relationship between implicit beliefs and habit missed the traditional significance threshold by a trivial margin. These findings support the assertion from habit theory that repeated past behavior is a key element in the development of habit, and, once developed, habit encourages future behavioral occurrences. Further, the data indicates a potential bi-directional relationship between habit and implicit beliefs, supporting the theory that implicit beliefs and habit may develop in tandem and over time mutually reinforce and strengthen each other. These findings provide an interesting early test of key assertions in habit theory. However, as the current study was limited by its modest sample size following attrition and use of self-reported measures, additional research is required to confirm and further investigate these effects.

## Research Data

sj-sav-1-hpq-10.1177_13591053231164492 – Supplemental material for Reciprocal relations between past behavior, implicit beliefs, and habits: A cross-lagged panel design Click here for additional data file. sj-sav-1-hpq-10.1177_13591053231164492 for Reciprocal relations between past behavior, implicit beliefs, and habits: A cross-lagged panel design by Kyra Hamilton, Daniel J Phipps, Natalie J Loxton, Kathryn L Modecki and Martin S Hagger in Journal of Health Psychology This article is distributed under the terms of the Creative Commons Attribution 4.0 License ( http://www.creativecommons.org/licenses/by/4.0/) which permits any use, reproduction and distribution of the work without further permission provided the original work is attributed as specified on the SAGE and Open Access pages ( https://us.sagepub.com/en-us/nam/open-access-at-sage).
